# Modular “Plug-and-Play” Photometer Based on IoT Digital Light Sensors: A DIY Approach for In Situ Analysis

**DOI:** 10.1021/acsomega.5c01857

**Published:** 2025-05-20

**Authors:** Diogo M. de Jesus, Felippe Delaqua, João Flávio da Silveira Petruci, Sidnei G. Silva

**Affiliations:** † Institute of Chemistry, 28119 Federal University of Uberlândia, 2121 João Naves de Ávila Avenue, Santa Mônica, Uberlândia MG38400-902, Brazil; ‡ Electrical Enginering Department, Federal University of Uberlândia, 2121 João Naves de Ávila Avenue, Santa Mônica, Uberlândia MG38400-902, Brazil

## Abstract

Portable photometers fabricated with low-cost optical components have been developed for a wide range of analytical applications. However, their analytical performance is closely related to the spectral range of the colored solution, as it depends on the detector material used in commercially available digital light sensors. A modular DIY, plug-and-play photometer was designed for quantitative absorbance measurements. Four digital light sensors (BH1750, TSL2591, LTR329, and LTR303) were evaluated for detecting colored solutions. LTR329 and LTR303 were found to provide optimal performance due to their broad spectral coverage, enhancing the device’s analytical capabilities. The photometer integrates an LED light source (635 nm) and a microcontroller (Adafruit ESP32-S3), enabling easy operation and data processing. It was employed to determine sulfide concentrations in wastewater using the methylene blue method and urea levels in Automotive SCR fluid samples with the Schiff reaction. The analytical results for sulfide (0.26–3.00 mg L^–1^) and urea (0.20–3.00% w/v) were comparable to those obtained by official methods, with detection limits of 0.09 mg L^–1^ for sulfide and 0.06% (w/v) for urea. The device’s modularity ensures easy component replacement, ensuring longevity and adaptability. With portability, 18-h battery life, and reliability, it is ideal for on-site analyses in remote settings. This study, not previously reported in the literature, presents the photometer as a valuable, sustainable tool for analytical measurements in resource-limited environments.

## Graphical abstract

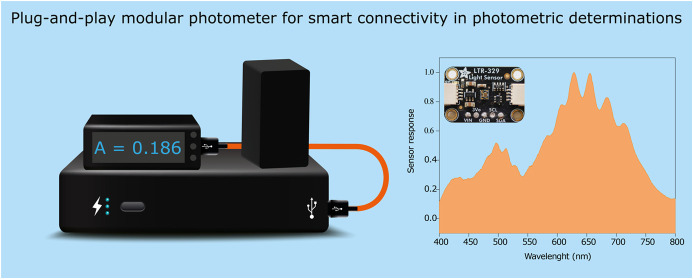

## 1 Introduction

Spectrophotometric-based methods have been widely employed for quantitative analysis in various fields, including environmental monitoring, clinical diagnostics, forensic investigations, and food analysis.^1^ Recently, the demand for on-site analysis has been becoming mandatory in a variety of scenarios, leading to the development and fabrication of portable (spectro)­photometers capable of collecting, analyzing, and transmitting the generated analytical data, enabling efficient real-time measurements.^2^ The integration of new technologies to enhance the portability of analytical optical instrumentation has been explored in the literature.^3–12^ The integration of 3D printing technology,^2,3,13–15^ microcontrollers,^12,14,15^ and communication between different devices for data processing and connectivity has garnered significant attention. Additionally, the incorporation of portable batteries further facilitates remote measurements, enabling researchers to conduct analyses directly at the sampling site.^4,6,11^ These advancements not only improve the portability and usability of analytical devices but also enhance their capabilities for real-time data acquisition and processing in field applications.

Digital-based portable photometers require low-power and miniaturized light sources, such as light-emitting diodes (LEDs), organic light-emitting diodes (oLEDS), and mini mercury lamps, and their application has been extensively evaluated and demonstrated in the literature. At the other end of the device, photodiodes, photoresistors, and even LEDs are usually employed as detectors, featuring their small size, affordability, and low energy consumption.^16^ The choice of the detector depends on the specific requirements of the analytical measurement, considering factors such as sensitivity, response time, dynamic range, and cost.^17^ Photodiodes typically offer higher resolution in terms of bits compared to photoresistors and LEDs, due to their ability to generate a proportional electrical current in response to light intensity variations, allowing for finer gradations in signal measurement. In contrast, photoresistors exhibit lower resolution and slower response times due to their nonlinear behavior under varying light intensities. This can lead to coarser measurements, and significant errors may occur if sufficient time for signal stabilization is not allowed.^18^ LEDs, when used as detectors, may also have lower resolution compared to photodiodes due to their lower photocurrents and shorter spectral responses.^16^ Therefore, in applications where high-resolution measurements are crucial, such as in precise spectroscopic analyses, photodiodes are often preferred. Recent literature features the expanding role of photodiodes as versatile detectors within analytical chemistry, particularly in photometric^6,14,19^ and fluorimetric^11,20,21^ analyses.

In addition to providing suitable analytical performance, the combination between LEDs and photodiodes can be easily connected with microcontrollers,^14,22^ enabling automated data acquisition and processing. Moreover, this compatibility aligns with the Internet of Things (IoT) concept, allowing for remote monitoring and data transmission, thereby enhancing the potential for on-field analysis.^23^ IoT has revolutionized how we interact with the world, and its influence is increasingly extending into analytical chemistry. The integration of IoT and analytical chemistry has driven the development of portable, low-cost, and do-it-yourself (DIY) devices designed for *in situ* applications.^13,23–25^ These innovations not only promote accessibility but also inspire creativity and exploration within the analytical chemistry community.

Nevertheless, the design of DIY photometers must carefully account for the spectral region, response time, and detection mode requirements to ensure optimal analytical performance for the intended application. However, a significant gap remains in the rational optimization of available components prior to their selection as light sources and detectors. In this study, we designed a modular DIY photometer and optimized the performance of several light sensors by evaluating the optical response of colored solutions within the electromagnetic spectrum.

## 2 Materials and Methods

### 2.1 Chemicals, Reagents, and Samples

All reagents used were of analytical grade and diluted in ultrapure water (18.2 MΩ·cm, Milli-Q Direct-Q3 system, Millipore, MA). Stock solutions of tartrazine yellow (Merck, Germany), potassium permanganate (Êxodo, Brazil), and methylene blue (Reagen, Brazil) were prepared by diluting appropriate amounts of the reagents in ultrapure water.

The determination of sulfide followed the procedure described by the Standard Methods for the Examination of Water and Wastewater.^26^ The stock solution of N,N-dimethyl-p-phenylenediamine oxalate (Neon, Brazil) was prepared by dissolving 0.27 g of the reagent in 10 mL of 50% H_2_SO_4_ (Dinâmica, Brazil). followed by 1:40 dilution in 50% H_2_SO_4_ (Reagent A). In a volumetric flask, 50 g of diammonium bifosphate (Cromoline, Brazil) were added to 100 mL of deionized water, and 100 g of FeCl_3_·6H_2_O (Dinâmica, Brazil) were dissolved in 40 mL of deionized water (Reagent B). The sulfide solution was obtained by dissolving crystals of Na_2_S.9H_2_O (Êxodo, Brazil) in deionized water and standardized via iodometry.^26^

For urea determination (99%, Vetec, Brazil), standard solutions containing the analyte were prepared by dissolving appropriate amounts of reagent in deionized water. For the preparation of the Schiff reagent, 0.8 g of dimethylaminobenzaldehyde (DMAB) (Vetec, Brazil) was dissolved in ethanol with 10% hydrochloric acid and the volume was completed to 50 mL (1.6% DMAB Sol). In this procedure, each prepared DMAB solution was used on the same day. For the quantification of urea, 1 mL of the DMAB solution was added to 9 mL of the sample or standard solution, followed by an expected reaction time of 2 min before the measurements.

### 2.2 Apparatus

Absorption and emission spectra were recorded using a multichannel spectrophotometer based on a CCD (Coupled Charge Device) array (USB 2000, Ocean Optics, Dunedin, FL). The wavelength response of the sensors and absorbance measurements were acquired using a spectrophotometer (FEMTO 600), following a procedure previously described in the literature.^4,27^ In this procedure, a 3D-printed cuvette holder support was utilized to enable the passage of radiation from the spectrophotometer and to serve as a mounting platform for the sensors. These sensors were assessed as detectors for spectrophotometric measurements. The support, replacing the original, ensured unobstructed light transmission while precisely positioning the sensor at a 180° angle relative to the light source. It was accurately installed in the original holder’s position. The spectrophotometer itself acted as the monochromatic radiation source, with signals collected by the sensors for each wavelength, recorded, and integrated into the sensor response graphs.

Four digital light sensors — BH1750, TSL2591, LTR329, and LTR303 (Adafruit) — were acquired to evaluate their analytical performance as detectors in the designed DIY photometer. All light sensors are composed of photodiodes that provide digital output, using 16 bits of A/D resolution. The correct selection of a sensor is key to defining the device capabilities in terms of spectral sensitivity and additional features that can be discussed according to its application. The BH1750 sensor can output data internally converted to lux units, but it offers few measurement setting options and has very low sensitivity at wavelengths below 470 nm and above 680 nm. The more advanced TSL2591 provides some measurement setting options, can output data in lux units, and includes three additional channels: infrared, visible, and full spectrum—the latter being a weighted sum of the other two. However, the TSL2591 showed poor signal response below 450 nm. The LTR329 sensor includes adjustable gain, integration time, and measurement rate, and demonstrated the widest response range across the visible spectrum among the tested sensors. Additionally, the light-dependent resistor (LDR), integrated via a simple module for microcontroller compatibility, operates in the most basic way. As expected from a fully analog sensor, the LDR does not allow digital adjustments and has limited sensitivity to shorter wavelengths in the visible spectrum.

An SMD (Surface Mount Device) LED (Sequins Ruby Red, Adafruit) emitting at 635 nm was employed as the light source for photometric measurements. This LED offers ease of implementation by allowing seamless integration into electronic circuits. Additionally, it operates at 3.3 V, a typical voltage used in ESP-32 boards and consumes a typical current suitable for such applications.

The Adafruit ESP32-S3 Feather Reverse TFT board was used as a microcontroller for the operation of the device components. This board incorporates a 240 × 135 TFT display positioned on the opposite face of the microcontroller, making it suitable for panel-mounted projects. Additionally, the board has three tactile buttons that function as an interface for interaction. The main features of the Adafruit ESP32-S3 Reverse TFT Feather include an ESP32-S3 Dual Core 240 MHz Tensilica microcontroller, with 4 MBytes of storage. The 1.14-in. colored IPS TFT screen provides a clear and colorful view from any angle. Three user tactile buttons (D0, D1, and D2), with the possibility of using D0/BOOT0 to enter the ROM bootloader mode, if necessary, are integrated. The tactile buttons were programmed for specific functionalities, such as saving the reference cell value for absorption calculations and changing the sensor gain. Work routines (firmware) were developed for each case and establish the set of sequential operations performed by the microcontroller. The Arduino Integrated Development Environment (Arduino IDE) was used to develop and compile the firmware, display the received raw values, and perform the systematic organization of each provided signal. The board offers power options via a universal serial bus (USB) type C or Lipoly battery, with integrated charging when powered by appropriate voltage and current.

### 2.3 Fabrication of the DIY Modular “Plug-and-Play” Device

A 3D printer (GTMax3D, Core A2v2, Brazil) was used to fabricate the photometer modules using polylactic acid (PLA) and acrylonitrile butadiene styrene (ABS) filaments. The light sensor and LED were positioned at 180° to enable transmittance measurements of the solution contained within a plastic cuvette. Moreover, a 5000 mA battery and the ESP-32 were accommodated into the device. The modular “plug-and-play” photometer is shown in Figure 1.

**Figure 1:**
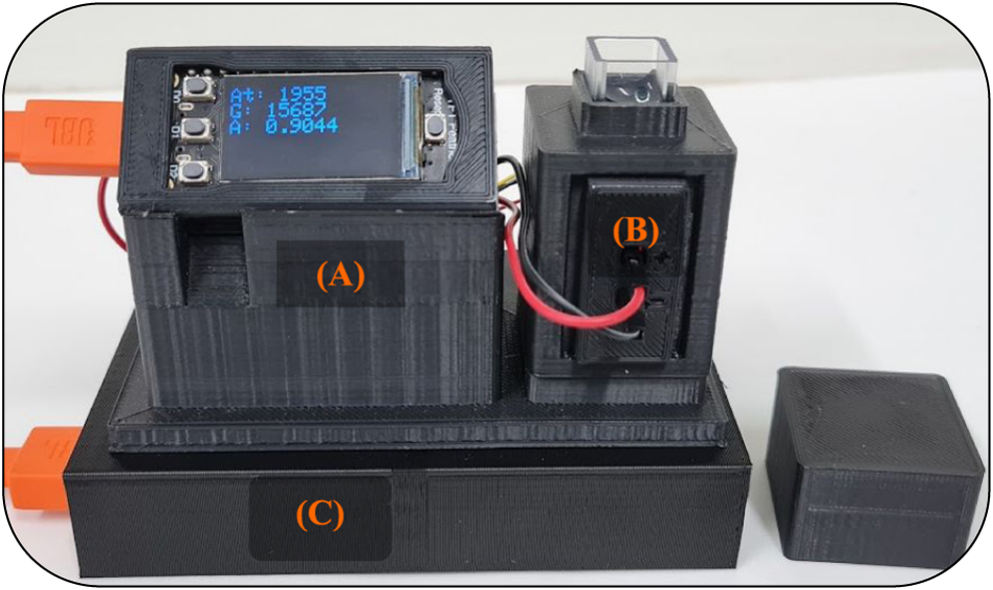
Photograph of the modular photometer setup (Figure). (A) ESP-32 microcontroller with integrated display. (B) Cuvette holder containing the 635 nm SMD LED (Sequins Ruby Red, Adafruit) as the light source and the sensor used as the detector. (C) Stand for the power bank, providing a portable energy supply.

### 2.4 Samples

The determination of urea was performed in two samples of Automotive Liquid Reducing Agent (sold as Arla-32 in Brazil, also known as AdBlue) obtained from local commerce in Uberlândia-MG (Brazil). The samples were prepared by dilution in deionized water (1:12). The method for the determination of sulfide was applied to three water samples collected from the Water Treatment Station in the city of Iturama and two samples from the Municipal Water and Sewage Department (DMAE) of Uberlândia. All samples were obtained directly from the outlet of the treatment reactors using a liquid sampler of the probe type. The aliquots for determination were aspirated from containers stored at 18 °C using a syringe and dispensed through a 0.45 μm hydrophilic polytetrafluoroethylene (H-PTFE) filter into plastic tubes. The samples were diluted 5-fold and analyzed using the proposed method. All the results were compared to those obtained by UV–vis spectrophotometry.

## 3 Results and Discussion

### 3.1 Light Sensors Response Range Evaluation

The development of a portable, modular photometer with IoT connectivity was the central theme of this work. In particular, the device is being developed using a DIY approach, which allows for easy component replacement and usability by individuals with limited experience in electronics and microcontrollers. Moreover, the device supports IoT communication, which enhances its flexibility for remote monitoring and data transmission. We evaluated four light sensors for their potential application as detectors in photometric measurements. The BH1750, TSL2591, LTR329, and LTR303 digital light sensors were identified as promising candidates based on their technical specifications, commercial availability, compatibility with microcontrollers, DIY philosophy, affordability, and compact size. For a sensor to serve as an effective optical light detector, certain key characteristics are essential: a broad response range in the visible region, an excellent signal-to-noise ratio, and robustness for consistent performance in varying conditions. Additionally, these sensors offer 16-bit resolution, enabling detailed measurements across a wide range of light intensities, which in turn allows for the detection of subtle changes.

To further investigate their suitability, an experiment was conducted to assess the spectral response range of each sensor. In this setup, the sensors were positioned in the cuvette holder of a commercial spectrophotometer, which served as the source of monochromatic radiation, for evaluating their performance.^4,26^ The response was measured from 400 to 800 nm, with increments of 1 nm. As shown in Figure 2, the results indicated that the LTR329 and LTR303 sensors provided comprehensive coverage across the entire evaluated spectrum (400–800 nm), while the BH1750 and TSL2591 sensors exhibited limitations, detecting lower wavelength ranges (below 460 and 425 nm, respectively). Therefore, for analytical applications in the visible region, sensors with broader spectral response, such as the LTR329 and LTR303, are more suitable. These sensors also meet the prerequisites of a DIY device and robustness, which are crucial for reliable performance, while still supporting IoT communication.

**Figure 2:**
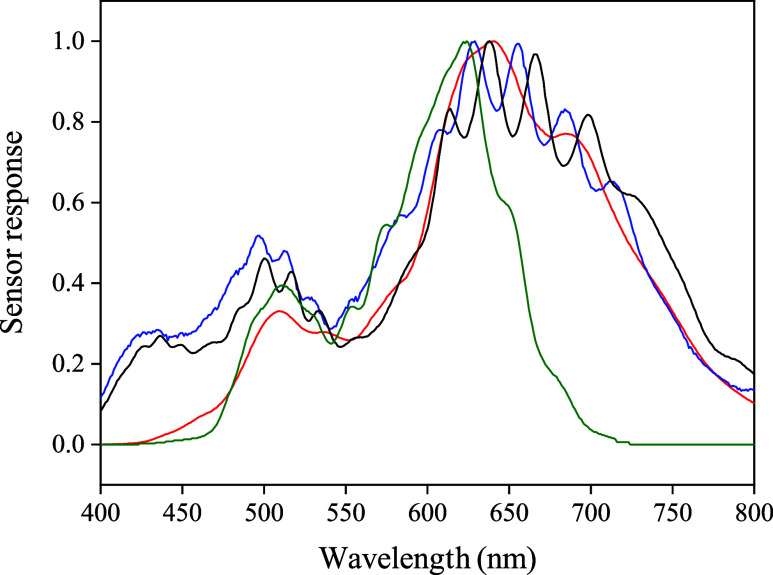
Effect of the spectral responses of optical sensors in the visible region of the electromagnetic spectrum. (Blue line) LTR 329, (Black line) LTR303, (Green Line) BH1750, and (Red line) TSL 2591.

### 3.2 Light Sensors Analytical Performances

To evaluate the analytical performance of the modular photometer, our investigation focused on assessing sensitivity, precision, and accuracy across the electromagnetic spectrum. To achieve this, we selected colored analytical solutions that collectively cover almost the entire visible spectrum, ensuring that the chosen substances have relevant analytical applications.

To begin our investigation, we replaced the detector of a commercial spectrophotometer with each of the sensors under evaluation, using the monochromatic radiation emitted by the spectrophotometer as the light source (Figure 3). Methylene blue, widely utilized in indirect determination methods for substances such as arsenic,^28^ hydrazine, and nitrite,^29^ was one of the substances evaluated. In addition, potassium permanganate, known for both direct and indirect determinations—especially in aqueous environments across diverse samples, including environmental^30^ and pharmaceutical^31^ contexts were evaluated. Furthermore, we included tartrazine, a widely used food dye,^32^ due to its relevance in food quality control and regulatory compliance, making it an important analyte in analytical chemistry. We obtained calibration curves for colored solutions that absorb in different regions of the electromagnetic spectrum, such as tartrazine (λ_max_ = 430 nm), potassium permanganate (λ_max_ 545 nm), and methylene blue (λ_max_ = 665 nm) and the results were compared to those obtained by the original detection system of the spectrophotometer. These analytical characteristics are presented in Table 1.

**Figure 3:**
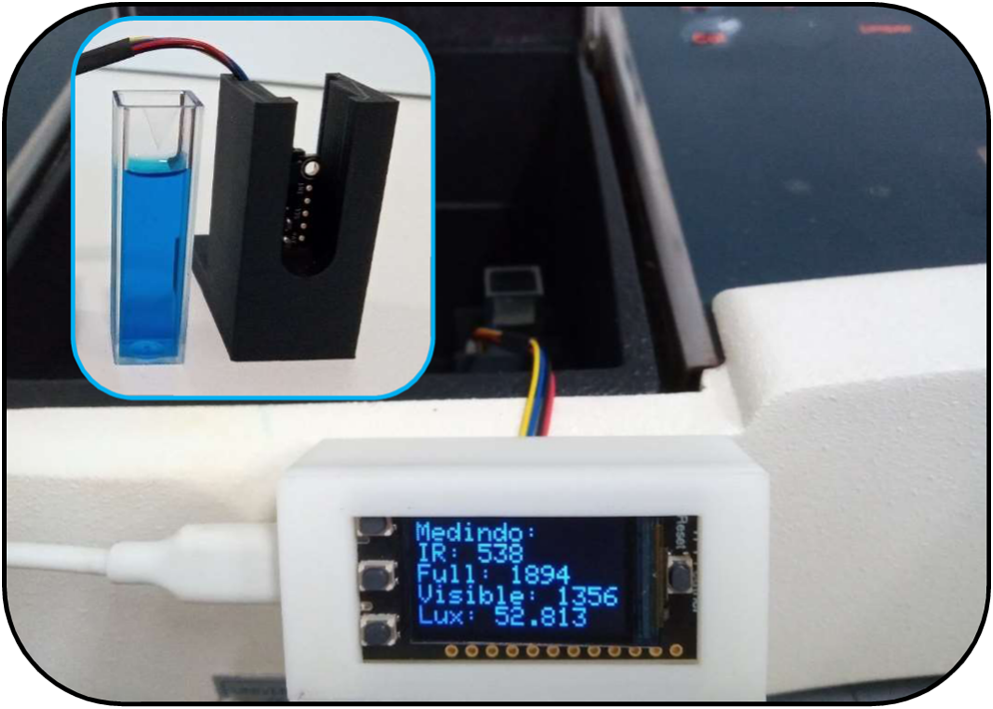
Setup photograph for evaluating sensor responses, showing a conventional cuvette and sensor placed inside a commercial spectrophotometer. Inset: close-up of a 3D-printed piece for holding the cuvette and sensor within the spectrophotometer.

**Table 1:** Analytical Characteristics of Different Detection Methods for Selected Analytes^a^

analyte	detection	linear equation	linear range (μmol L^–1^)	*R* ^2^	LOD (μmol L^–1^)
tartrazine yellow	TSL	*A* = 0.001 + 5.74 × 10^–3^ *C*	1.0–50.0	0.958	0.81
	LTR	*A* = 0.001 + 6.48 × 10^–3^C	1.0–70.0	0.995	0.13
	UV–vis	*A* = 0.001 + 6.50 × 10^–3^ *C*	1.0–70.0	0.996	0.32
KMnO_4_	TSL	*A* = −0.010 + 1.77 × 10^–3^ *C*	5.0–400.0	0.997	0.90
	LTR	*A* = 0.010 + 1.76 × 10^–3^ *C*	5.0–400.0	0.996	0.50
	UV–vis	*A* = 0.010 + 1.75 × 10^–3^ *C*	5.0–400.0	0.997	1.20
methylene Blue	TSL	*A* = 0.040 + 0.0581*C*	0.10–25.0	0.993	0.03
	LTR	*A* = 0.001 + 0.0599*C*	0.10–25.0	0.997	0.02
	UV–vis	*A* = 0.110 + 0.0484*C*	0.10–25.0	0.994	0.04

a Linear equations, linear ranges, coefficient of determination (*R* ^2^), and limit of detection (LOD) values are provided for each analyte and detection method.

For all sensors, the raw output value is used to estimate the transmittance *T* of the solution according to the eq 1.

1 $$T=\frac{I}{I_{0}}$$

where *I* is the value measured on a sample solution and *I*_0_ is the value measured on a reference solution. The absorbance *A* is obtained using the eq 2.

2 A=−log⁡T

The BH1750 sensor was not included in the evaluation due to its shorter spectral response compared to the other sensors. Due to the close similarity in spectral responses between the LTR329 and LTR303 sensors, we opted to assess only the LTR329. When comparing the sensitivities of the sensors for different analytes, it was observed that, in general, they presented very similar values, indicating consistency in their performances. However, specifically for the detection of tartrazine, the sensitivity observed when the TSL2591 sensor was used as a detector was lower compared to that of the LTR329 sensor and the commercial spectrophotometer. The TSL2591 sensor reached a detection limit similar to that obtained by the spectrophotometer, unlike the other dyes, whose calculated detection limit values were lower than those of the spectrophotometer. The LTR329 sensor showed satisfactory performance in terms of sensitivity and linearity in the three spectral ranges.

The selection of the reaction between urea and dimethylaminobenzaldehyde (DMAB) for urea determination was deliberate, considering the spectral response variation among sensors, particularly pronounced within the 400–450 nm wavelength range. This choice aimed to monitor this spectral region precisely, aligning with the absorption peak of the resulting Schiff base product (Figure 4), which manifests as a yellow hue (λ_max_ = 420 nm).^33,34^ Among the sensors assessed, the LTR329 exhibited superior responsiveness within this critical spectral band, offering enhanced sensitivity compared to its counterparts. Consequently, the analytical efficacy for urea determination in Automotive Liquid Reducing Agent (Arla-32) samples was scrutinized, bearing in mind the standard urea concentration in commercial compositions, typically around 32% (m/v).

**Figure 4:**
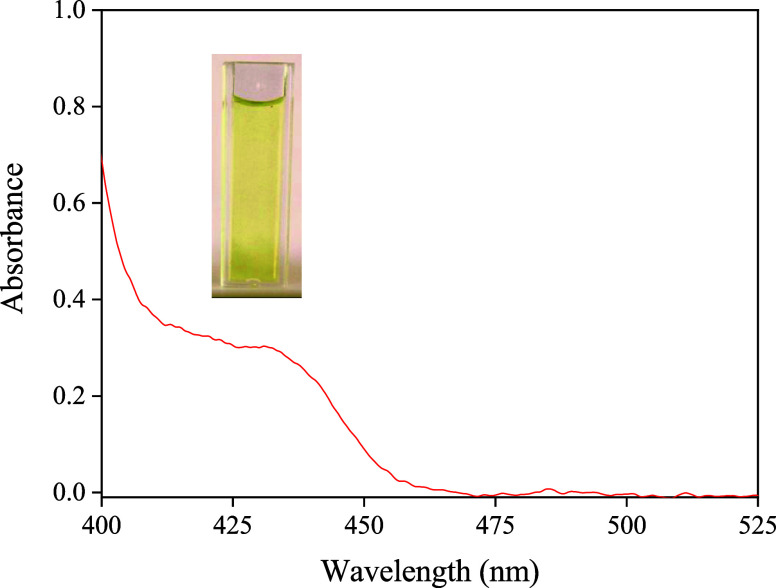
Spectrum of the Schiff base product obtained from the reaction between urea and dimethylaminobenzaldehyde (DMAB). A photograph of the reaction product inside a 1 cm path length cuvette is embedded in the graph for visual reference.

Analytical characteristics were evaluated for urea determination using the Schiff method with the LTR329 sensor as a detector, comparing results with those obtained from the embedded detector of a commercial spectrophotometer. In both cases, the monochromator source was the same, a Femto600s native tungsten and deuterium lamp, and the desired wavelength, selected through the spectrophotometer wavelength selection handle.

The LTR329 sensor exhibited a linear equation (*A* = 0.062 + 0.298*C*) across a concentration range of 0.20–3.00% (w/v), achieving an *R* ^2^ of 0.998 and the detection limit (3.3σ/*S*) of 0.06% (w/v). In comparison, the use of the commercial spectrophotometer showed a similar linear equation (*A* = 0.066 + 0.306*C*) over the same concentration range, with an *R* ^2^ of 0.998 and an LD of 0.07% (w/v).

These results underscore the comparable performance of both the LTR329 sensor and UV–vis spectrophotometer in accurately determining urea concentrations. Meanwhile, for UV–vis (Femto 600-s), the linear equation is *A* = 0.066 + 0.306*C*, covering the same linear range of 0.20–3.00% (w/v), with an *R* ^2^ of 0.998 and an LOD (3.3σ/S) of 0.07% (w/v). These data underscore the comparable performance of both the LTR329 sensor and the UV–vis spectrophotometer in accurately determining urea concentrations, reinforcing the reliability of the LTR329 sensor in this analytical application. The similarity between the results can be attributed to several fundamental reasons. First, the chosen reaction generates a product that absorbs light in the electromagnetic spectrum region where the LTR329 sensor demonstrated a satisfactory spectral response, between 400 and 450 nm. This compatibility between the absorption region of the formed product and the efficient response range of the sensor significantly contributes to the similarity of the results.

To further evaluate the accuracy of the urea determination method using the Schiff reaction, two samples of Automotive Liquid Reducing Agent (Arla-32) from local commerce were analyzed. The samples were prepared by diluting Arla aliquots 12-fold with deionized water. Subsequently, the results were compared between those obtained with a photometer equipped with the LTR329 sensor and conventional UV–vis spectrophotometry. For sample A1, the photometer yielded a calculated urea concentration of 32.97 ± 0.67%, while UV–vis yielded 33.6 ± 1.01%. In the same way, for sample A2, the photometer recorded 33.9 ± 0.70%, and UV–vis gave 35.28 ± 1.1%. Overall, the comparison showed that the urea determination results for both samples were consistent across the LTR329 sensor and UV–vis spectrophotometry methods.

### 3.3 Fabrication of the Modular DIY Photometer and Application in Real-World Samples

Based on the superior analytical performance over a wide spectral range, the modular DIY photometer was built using the LTR329 sensor as a detector. This property not only demonstrates the sensor’s effectiveness but significantly enhances the device’s applicability in quantitative analyses. The study focused on evaluating the device for sulfide determination using the methylene blue method, which determines the concentration of sulfide in solution.

Based on these considerations and with the aim of building a portable device for on-site analysis, an SMD LED (λ_max_ = 635 nm) was used as the light source for absorbance measurements. The LED chosen was the Sequin LED from Adafruit, which has the advantage of being powered directly by the ESP32 board, with a voltage compatible with the microcontroller’s output (3.3 V). Additionally, the photometer employs a 5000 mAh battery to power the microcontroller. Supports for each of the components, including the sample holder, detector, LED, and ESP-32, were 3D printed. Figure 5 displays the spectrum of the methylene blue solution overlaid with the emission spectrum of the SMD LED used as the light source in photometric measurements employing the proposed photometer.

**Figure 5:**
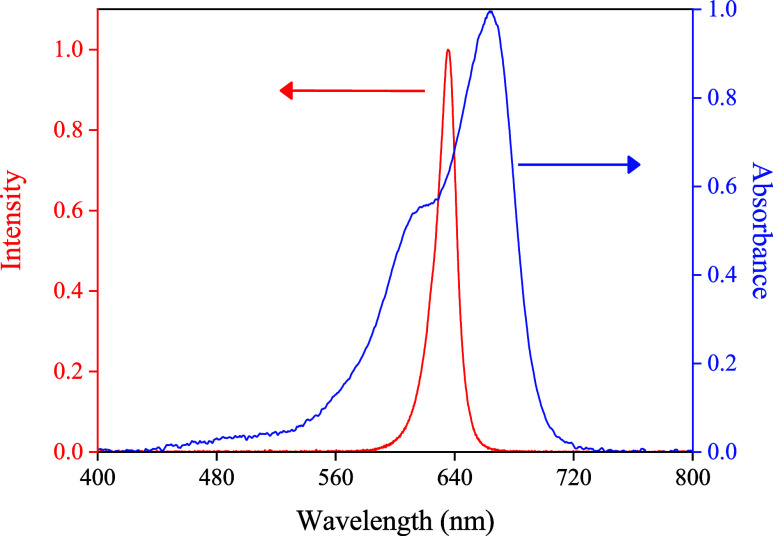
Spectrum of emission from the LED (λ_max_ = 635 nm) used as the light source (red line), overlaid with the absorption spectra of methylene blue (blue line).

The analytical characteristics of the proposed photometer were compared with UV–vis spectrophotometry. For the photometer, the linear equation was determined as *A* = 0.042 + 0.376*C*, with a linear range of 0.10–3.00 mg L^–1^ (*R* ^2^ = 0.996) and a detection limit (3.3σ/S) of 0.08 mg L^–1^. In contrast, UV–vis spectrophotometry exhibited a linear equation of *A* = 0.014 + 0.494*C*, covering a linear range of 0.05–1.00 mg L^–1^ (*R* ^2^ = 0.997) and an LOD (3.3σ/S) of 0.17 mg L^–1^. These results underscore the performance of the proposed photometer, demonstrating comparable analytical characteristics to UV–vis spectrophotometry in the determination of methylene blue concentrations.

The stability of the proposed photometer’s analytical signal was carefully evaluated in an experiment monitored for 600 min, where the sensor was exposed to LED light. This evaluated time corresponds to the autonomy of the battery used to power the photometer, which was estimated at 18 h. The observed variation in the signal, in the form of relative standard deviation, was 0.24%. These findings highlight the consistent variation observed in the LED signal, even when powered by a lithium battery. This consistency underscores the repeatability of the LED-based system, suggesting its reliability for repeated measurements. This result demonstrates the efficiency of the proposed device, which, by integrating the LTR329 sensor and an appropriate LED, emerges as a promising tool for qualitative and quantitative analytical applications.

It was observed that the sensitivity of the proposed method was approximately 24% lower compared to a commercial UV–vis spectrophotometer. Although the characteristics showed a slight inferiority compared to the official method, the proposed system offers the advantage of allowing in situ determinations, resulting in a faster response. Additionally, the ability to transmit signals autonomously over the Internet is an additional feature of the system. This functionality contributes to the method’s practicality and efficiency, highlighting its applicability in environments where autonomy and agility in obtaining results are priorities.

The determinations of the obtained samples are shown in Table 2. The results obtained were consistent with the paired *t* test at 95% confidence (critical *t* = 2.776 and calculated *t* = 1.549; (*n* = 5)), i.e., the measurements obtained for the portable system and spectrophotometer statistically refer to the same mean. Data analysis can confirm the device’s reliability and attest to its ability to provide accurate and consistent results for sulfide determination in real water samples from different treatment plants. The variance between the measurements, found for the portable system, must refer to the reduced structure and the inevitable movement between the photometer parts, being acceptable for on-site sample collection. Measurements obtained from calibration curves provided a coefficient of variation of 4.77 at intermediate concentration points.

**Table 2:** Sulfide concentrations (mg L^–1^) determined by the proposed method and UV–VIS spectrophotometry in water samples collected at the Iturama Water Treatment Plant and the Municipal Water and Sewage Department (DMAE) in Uberlândia^a^

sample	proposed method	reference	recovery (%)
A1	6.1 ± 0.2	5.6 ± 0.1	108.9
A2	3.7 ± 0.2	3.7 ± 0.1	100.0
A3	3.1 ± 0.3	2.8 ± 0.1	110.7
B1	2.0 ± 0.1	1.7 ± 0.1	117.6
B2	1.5 ± 0.1	1.5 ± 0.1	100.0

a The samples were diluted 5 times to fit within the linear range.

Based on its superior analytical performance across a broad spectral range, the modular DIY photometer was constructed using the LTR329 sensor as the optical detector. This choice not only demonstrates the sensor’s effectiveness but also significantly expands the device’s applicability for quantitative analyses. Compared to conventional benchtop spectrophotometers, the developed system offers several advantages, including compact size, low energy consumption, and true portability, making it particularly suitable for in situ measurements. The device operates on a rechargeable power bank, ensuring extended autonomy and eliminating the need for a constant power supply. Additionally, the modular structure allows for easy replacement of LEDs, enabling rapid adaptation to different colorimetric methods. Wireless communication via Wi-Fi further enhances its usability in field applications and remote monitoring. While LED replacement is required for specific applications due to the use of discrete light sources, this trade-off is compensated by the low cost, versatility, and ease of maintenance of the system. To demonstrate its analytical potential, the photometer was evaluated for sulfide determination using the methylene blue method, a well-established technique for quantifying sulfide concentrations in aqueous solutions.

## 4 Conclusions

The LTR sensors demonstrated excellent spectral performance, covering a broader portion of the visible region of the electromagnetic spectrum. This feature significantly enhances the device’s analytical capabilities, making it suitable for a wide range of quantitative and qualitative analyses within the visible spectrum. The study of the sensors’ spectral response is crucial for selecting the most appropriate sensor for spectrophotometric measurements. Moreover, the photometer developed in this study offers easy component replacement, ensuring both longevity and adaptability to diverse analytical needs. Its portability (11 × 7 × 10 cm and 250 g) and extended battery life (over 18 h) further contribute to its practicality, particularly for on-site analyses in remote or field settings. The device is low-cost and could obtain statistically equivalent results as a benchtop spectrophotometer. The so-called analytical performance-price balance is present here — the device is vulnerable to spectral interference due to the absence of a dedicated light source and relies on LEDs to maintain low cost and simplicity, which can often lead to loss of exactness in more complex samples, such as wastewater. Even so, the device showed sufficient performance for analytical applications. In general, this demonstrates the device’s reliability and precision, positioning it as a valuable tool for various applications in analytical chemistry.
